# A prospective pilot study of analysis of surgical margins of breast cancers using high-resolution sonography

**DOI:** 10.1186/s40064-016-1921-2

**Published:** 2016-03-01

**Authors:** Anabel M. Scaranelo, Hadas Moshonov, Jaime Escallon

**Affiliations:** Division of Breast Imaging, Joint Department of Medical Imaging, University Health Network, Mount Sinai Hospital and Women’s College Hospital, University of Toronto, 600 University Avenue, Toronto, ON M5G 1X5 Canada; Research Office of The Joint Department of Medical Imaging, University Health Network, Mount Sinai Hospital and Women’s College Hospital, University of Toronto, Toronto, ON Canada; Surgical Oncology Department, Marvelle Koffler Breast Centre, Mount Sinai Hospital, University of Toronto, Toronto, ON Canada; Princess Margaret Cancer Centre, 610 University Avenue, Rm 3-922, Toronto, ON M5G 2M9 Canada

**Keywords:** Breast cancer, Specimen, Margins, Sonography

## Abstract

To investigate the role of high-resolution specimen sonography (SS) to determine the precise location of the targeted lesion in relation to the six surgical margins; the specimen digital radiography isocenter and the correlation with the rate of re-excision and residual tumour. Freshly excised surgical specimens were scanned by a breast radiologist using a high-frequency linear transducer in a cohort of 25 consecutive women undergoing breast conservation. Sonographic measurements of radial distances from all six margins (superior, inferior, lateral, medial, anterior and posterior) were obtained. Sonographic positive margin status was defined as targeted mass identified <5 mm from the tissue edge. The paired *t* test was used for statistical comparisons between sonographic and pathological measurements. The median cancer size was 15 mm (range 3.80–42 mm; 95 % CI 9.8–18) on sonography and 16 mm (range 2–60 mm; 95 % CI 15–20) on surgical pathology. SS showed 100 % sensitivity and 59 % specificity in the evaluation of surgical pathology margins. 20 % (5 of 25) patients had positive margins where 60 % were in situ carcinoma. The likelihood of carcinoma at the initial surgical margins was significantly higher in dense breasts (3/6 = 50 % vs 1/17 = 5.8 %; p = 0.04). The deviation of the isocenter of the specimens was found not significant. SS is a valuable tool for identify the cancer within the specimen, and better asses the margins. It is of significant importance in patients with dense breasts where specimen radiography is of limited value.

## Background

Breast-conserving surgery (BCS) is the first option for women with early stage disease or in some more advanced cases could be considered after neo-adjuvant chemotherapy is completed (Fitzal and Gnant 2006; NIH Consensus Statement 1990; McCahill et al. 2012). The major concern after BCS is the risk of local recurrence and several factors like patient’s age, tumour extension, axillary node status, histological type, hormonal receptors expression, and surgical margins have been studied (Botteri et al. 2010; Nottage et al. 2006; Di Saverio et al. 2008).

BCS followed by postoperative radiation shows data similar to mastectomy when comparing long-term survival for early stage breast cancers (Veronesi et al. 2002; Fisher et al. 2002). A successful surgical procedure implies, a complete excision of the entire malignancy surrounded by a margin of normal breast tissue. Historically, preoperative localization of breast cancer was performed by inserting a hook-wire-needle in the lesion under mammographic guidance in the radiology department (Fornage et al. 1994). To avoid discomfort to patient (breast compression) and because majority of breast cancers can be visualized under ultrasound, the needle-localization has been performed under sonographic guidance for palpable or non-palpable tumours. Consequently it is expected that the post excision sonography in addition to the post excision digital specimen radiography could be performed to document that the targeted lesion was excised with clear margins.

The majority of women with a newly diagnosed breast cancer that presents with early stage have a potentially curable disease and therefore the knowledge by the surgeon of the precise location of the cancer in the excised surgical specimen is important as well the distance from the edges of the specimen. Several methods have been proposed for margin assessment and the gold standard is based on the surgical pathology results of the lumpectomy specimen with a spatial orientation with multicolour inking (Gibson et al. 2001; Angarita et al. 2014). However those results are not ready during the time of surgery and the microscopic extent of breast cancer is not possible to discern with naked-eyes by the surgeon. Furthermore, lumpectomy with positive margins and a second surgery (re-excision) where no residual tumour was found on surgical pathology demonstrates 35–49.5 % of patients receiving unnecessary re-excision (Luu et al. 1999; Dooley and Parker 2005; Cao et al. 2005; Scopa et al. 2006). The current available high-resolution sonography in the everyday state-of-the-art sonographic machines has been shown to be accurate in visualizing small as 1–2 mm breast masses. For this reason, we would like to investigate the role of high-resolution sonography to determine the precise location of the targeted lesion in relation to the six surgical margins and the specimen radiography isocenter of the specimen and the correlation with the rate of re-excision and residual tumour.

## Methods

### Study design and patient population

This pilot study was a prospective, non-randomized, single arm, control trial involving two institutions, academic tertiary hospitals in Canada. Specimen radiograph followed by specimen sonography was being performed as part of the routine care at our institution to demonstrate the presence of the targeted lesion. Thus, our institutional ethics board considered specimen sonography with measurements as innovative care and did not require additional approval for its use. Inclusion criteria included women over 18 years with nonpalpable intraductal or invasive breast cancers. All patients had signed consented for BCS and underwent image guided pre-operative needle localization in the same day of the breast surgery. Patients that were pregnant or lactating and the ones who received new adjuvant chemotherapy were excluded.

### Specimens

**Fig. 1:**
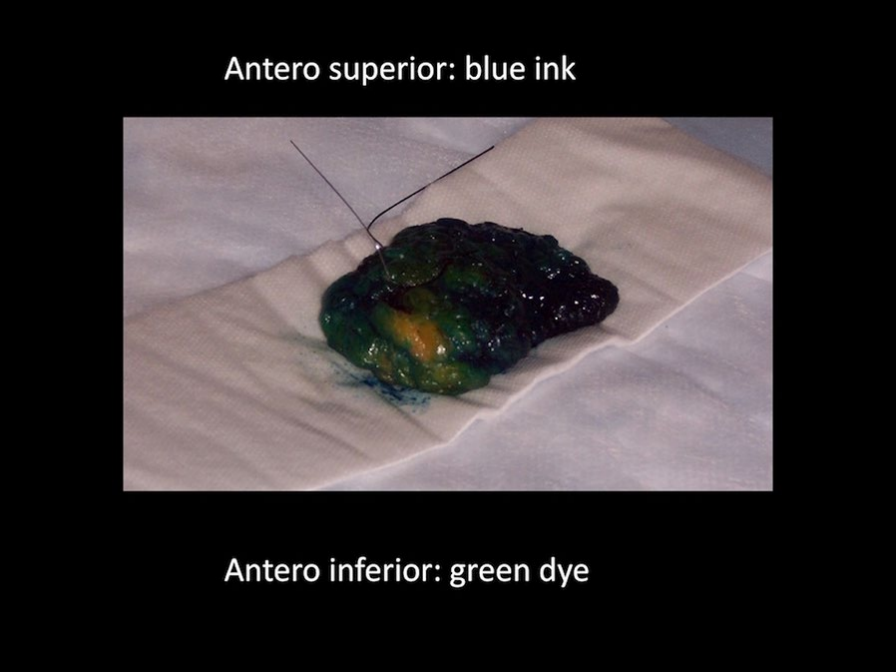
Photograph of an intra operative breast specimen with the inked edges: *blue* ink used to demonstrate the anterior–superior margin and *green* ink to stain the anterior–inferior margin. In addition, a short-stitch is placed by the surgeon on the anterior–superior margin and a long-stitch is left to demarcate the lateral margin

**Fig. 2:**
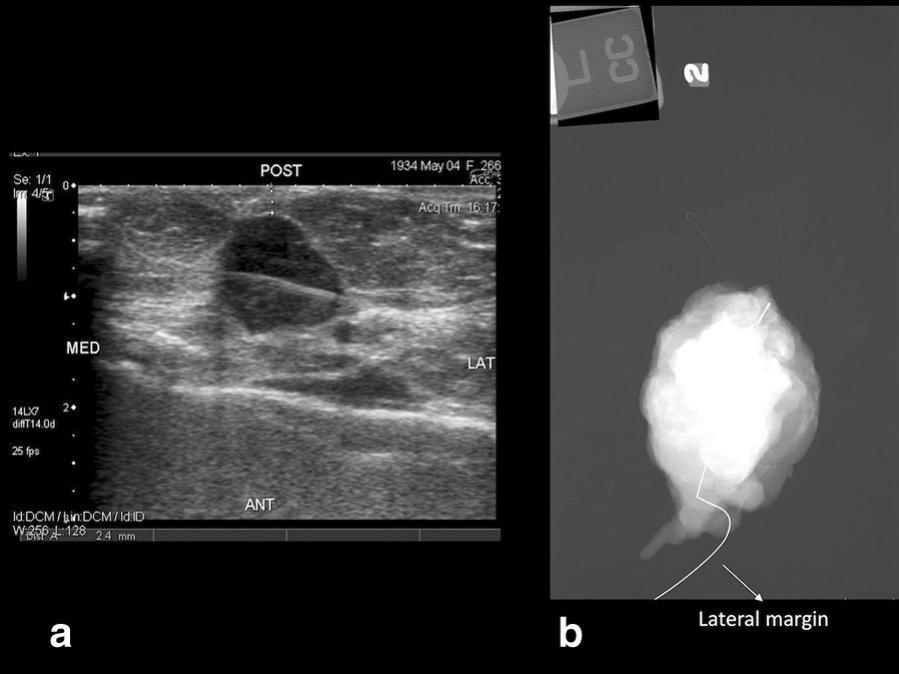
**a** Sonographic imaging of an intra operative breast specimen with identification of the targeted breast cancer. **b** Digital radiography of the same specimen shows dense tissue without identification of the cancer

All peri-operative specimens were spatial oriented using suture (e.g. long suture for lateral, short suture for superior) by the attending surgeon. After the imaging assessment by the attending radiologist, the specimens received dual ink markings (e.g. green dye for antero inferior, blue dye for antero superior) by the attending pathologist (Fig. 1). A single breast radiologist (AMS) with 19 years of experience in breast ultrasonography was enrolled to reduce interobserver variation. The specimen was scanned in two dimensions: first in antero-posterior and then flipped and re-scanned in postero-anterior (Fig. 2a) with a small amount of saline on top of it as a contact media without having to use fluid immersion (Fornage et al. 1994). The targeted lesion was identified and six radial distances from all margins (superior, inferior, lateral, medial, anterior and posterior) were recorded. The radial distances were defined as the distance between the hypo echoic mass to the edge of the specimen. Then a digital radiograph of the specimen (Fig. 2b) was obtained oriented by a breast technologist with the standard technique that demonstrates the long suture stitch in the lateral margin associated to the identification of the digital radiograph side (right or left) using radiopaque (plumb) medical imaging letter set. All specimens radiographs were reviewed by an independent radiologist without correlation with the specimen sonogram measurements. Specimen measurements were recorded using specimen radiograph for largest dimensions medio-lateral (ML) and supero-inferior (SI) and sonography for largest antero-posterior (AP) dimensions and volume was estimated using the equation: Volume = ML × SI × AP × k, where k is a constant (k = 0.52333). The specimens were evaluated by board certified pathologists who were blinded to this study that were sub-specialized with more than 15 years of experience in breast pathology each one. The specimen radiograph isocenter was defined as the point of intersection of ML/2 and SI/2 and the deviation from this point was recorded for ultrasound and surgical pathology measurements.

### Medical imaging devices

A full-filled digital mammography unit (Senographe 2000D; GE Medical Systems, Milwaukee, WI, USA) was used in all examinations. The equipment used to perform sonography of the specimen included a linear transducer of Aplio series (Toshiba America Medical Systems) with a frequency range of 7.2–18 MHz.

### Statistical analysis

The margins analyzed were categorized into six main groups: superior, inferior, lateral, medial, anterior and posterior and sub categorized into two groups: negative and positive margins by sonography and correlated to pathology as the gold standard. For the purpose of this study, a positive sonographic margin was considered to be the hypoechoic targeted mass identified <5 mm from the inked edge of tissue. Positive pathologic margins were considered when the invasive cancers without extensive DCIS were detected at the edge of tissue and or then DCIS were found <1 mm from the inked margin on microscopy. We also evaluated the distance from margin to sonographic target cut-off of 2 mm and compared with surgical outcomes. Continuous variables were described using mean ± SD or median and range; categorical variables using frequency and percentage. Statistical comparisons between sonographic and pathological measurements were performed using the paired *t* test. The sensitivity, specificity, positive and negative predictive values were calculated for predicting positive histological margins. Statistical analysis was performed using SPSS software (Version 20, IBM SSPS, Chicago, IL, USA) and a *p* value of 0.05 or less was used to determine the statistical significance.

## Results

A total of 25 consecutive women were recruited. Table 1 shows the characteristics of the study population. The mean age was 62 years (range 37–85 years). 11 of 25 women (44 %) presented with a palpable breast lump. All patients had pre operative needle localization under sonographic guidance of 27 targeted breast cancers. Invasive lobular carcinoma was found in FOUR cases (15 %), where three were not associated to any other invasive cancer and one was mixed with invasive ductal carcinoma (IDC). All others 23 targeted lesions (85 %) were IDC, where 5 of 23 (22 %) were multifocal cancers. The association with ductal carcinoma in situ (DCIS) was present in 19 of 27 (70 %) lesions. Nuclear Grade 2 was the most frequently identified, present in 14 of the 27 cancers (52 %), followed by eight cases with Grade 3 (30 %) and five cases with Grade 1 (18 %).

**Table 1:** Study population characteristics

Histopathology	Number of patients	Mean patient age (years)	Number of specimens	Number of hook-wired lesions	Mean specimen volume (ml)
IDC	5	71	5	5	58
IDC + DCIS	13	57	13	13	66
ILC	3	71	3	3	79
Mixed IDC + ILC	1	59	1	1	90.8
Multifocal IDC	3	55	3	5	120
Total	25	61	25	27	76.6

*DCIS* ductal carcinoma in situ, *IDC* invasive ductal carcinoma, *ILC* invasive lobular carcinoma

Specimen volumetric measurements using imaging methods ranged from 13.36 to 265.84 cc, median 55.91 cc (95 % CI 42.14–70.4) and using pathology ranged from 23.55 to 200 cc, median 65.86 cc (95 % CI 44.93–90.82). In ten cases the measurements obtained by imaging methods were higher than pathology macroscopic measurements; the mean difference was estimated to be 1.97 (95 % CI −14.1 to 18.03). However, it did not reach statistical significant (p = 0.803, paired *t* test).

Specimen sonography identified 100 % of the targeted lesions and the wire was found present in all the specimens. The median cancer size using imaging methods was 15 mm (range 3.80–42 mm; 95 % CI 9.8–18) on sonography and 16 mm (range 2–60 mm; 95 % CI 15–20) on surgical pathology. Fifteen cancers had sonographic size smaller than pathology, seven cases the underestimation was between 1 and 5 mm and eight cases more than 5 mm. In nine cases the sonographic largest dimension was considered higher than the ones obtained by pathology, the median of difference in measurements was 1.5 mm (range 0.2–15 mm). Specimen sonography (hypoechoic targeted mass found <5 mm from the edge) had a sensitivity of 100 % and specificity of 59 % for evaluation of surgical pathology margins in our cohort (Table 2). If a cut-off of 2 mm was used the sensitivity would be of 20 % and specificity of 86 %.

**Table 2:** Comparison of specimen sonography margins with surgical pathology

	Surgical pathology	Margins report	Total
	Positive	Negative	
Specimen sonography			
Positive	4 (true positive)	10 (false positive)	14
Negative	0 (false negative)	13 (true negative)	13
Total	4	23	27

11 of 25 patients (44 %) had additional margins excised by surgeon’s decision in the day of surgery, and 5 (20 %) cases had positive margins where three were in situ carcinoma. Of those five patients with positive margins, three were found uninvolved by cancer with the additional margins excised during the same surgical time and two patients had a mastectomy in another day. Therefore of 25 patients with a mean 6.2 years of follow-up, 2 (8 %) had a mastectomy and one (with negative margins) had another lumpectomy for an additional cancer with <3 years follow-up. The likelihood of carcinoma at the initial surgical margins was significantly higher in dense breasts (ACR densities “c” and “d”) when compared to the ones mildly dense or fatty (3/6 = 50 % vs 1/17 = 5.8 %; p = 0.04) No significant difference in the frequency of the cancer at the margins was identified as a functions of sonographic lesion size, clinical presentation (palpable or not) or volume of the specimen excised (Table 3).

**Table 3:** Features associated with presence of in situ or invasive carcinoma at surgical specimen margins

Feature analysed	Number of cases with cancer at margins (%)	p value
Breast density		
Density “c” + density “d”	3/6 (50)	0.05
Density “a” + density “b”	1/17 (5.8)	
Clinical presentation		
Palpable lump	2/14 (14.2)	0.68
Non-palpable	2/9 (22.2)	
Lesion size on US		
<1.5 cm	2/10 (20)	0.48
≥1.5 cm	2/13 (15.3)	
Volume of specimen		
<50 ml	1/10 (10)	0.80
≥50 ml	3/13 (23)	

The deviation of more than 1 cm from the in plane isocenter in the ML (lateral margin and medial margin) view of the specimens was found not significant (p = 0.301, paired *t* test), however when we analyzed the deviation of more than 1 cm from the isocenter in the SI (superior margin and inferior margin) plane, we observed that all positive margins had a deviation that ranged from 11 to 26 mm from the isocenter of the specimen (p = 0.0565, paired *t* test). The average deviation from the in plane isocenter in the ML (lateral margin and medial margin) sonographic view of the specimens was found to be smaller than the corresponding deviation based on pathology (11.88 vs 14.25 respectively). However, the difference did not reach statistical significance (p = 0.639, paired *t* test). Further, there was no significant difference in the average deviation from the in plane isocenter in the SI (superior margin and inferior margin) view between sonographic and pathological measurements (14.16 vs 14.84 respectively, p = 0.828, paired *t* test).

## Discussion

Our results demonstrates that specimen sonography was able to identify all targeted cancers with a high sensitivity to demonstrate that margins that are imaging free with a distance of at least 5 mm safety are also without cancer cells when compared with the pathologist evaluation. Studies (Fornage et al. 1994) that described the sonographic technique of scanning excised breast specimens were performed with equipments with imaging resolution far below of what is the state of the art in the current market.

The presence of carcinoma in the first excised surgical margins on this cohort was 20 % (5 of 25) and all cases were demonstrated by the specimen sonogram. Of the five patients with positive pathology margins, one underwent to mastectomy in another day and 4 (80 %) had additional margins excised by surgeon during the same surgical appointment and none were involved by invasive carcinoma and in just one patient the additional margins were involved by DCIS. Presence of residual carcinoma on re-excision are reported in 29–45 % of patients (Cao et al. 2005; Scopa et al. 2006). The estimated rate of residual carcinoma on re-excision after using the sonographic technique in this cohort went from 40 % (2 of 5) to a corrected rate of 8 % (2 of 25) because one patient had re-excision in another day. From patient’s perspective there is anxiety and stress to undergo additional surgery despite the fact that this is under studied in the literature.

This study describes an innovative way to measure the peri operative fresh specimen determining the in plane isocenters (equidistant point from the ML and SI planes) and establishing the relationship of this point with the pathology measurements that are obtained after fixation of the specimen. Such correlation was not demonstrated yet in the literature search of the last 30 years. The cases that had a high deviation from the isocenter in the ML plane demonstrated a trend to be the ones with positive margins, the cases with high deviation in the SI plane did not show similar findings. Some would argue that a target that is not well positioned in the isocenter may be close to one of the margins and therefore with a higher probability to have a positive surgical margin. The number of positive margins however in this pilot study was small to reach statistical significance to evaluate this observation. The volumetric measurement of the fresh excised specimen using both digital mammography and sonography was higher than pathology macroscopic measurements that is measured without fixation in 10 of 25 cases. This was prevalent in the larger specimens above the median (55.91 cc) that had associated areas of calcifications. This may be associated to variability of human factor as several technicians that were involved did not have any specific training to measure the fresh specimen in a standardized manner for this study. Nevertheless, the median cancer size 15 mm on imaging methods and 16 mm on pathology was not discrepant.

A metanalysis of the surgical margins on local recurrence (Houssami et al. 2010) or other studies evaluating surgical margins (Botteri et al. 2010; Nottage et al. 2006; Scopa et al. 2006) did not describe association with breast density; however we noted that positive initial surgical margins were significantly higher in dense breasts (50 vs 5.8 %) in this cohort. As sonography has better conspicuity to discriminate two points when scanning dense hyper echoic tissue than fatty breasts, it is expected that the use of specimen sonogram would be of importance as none of the cancers were missed on sonography.

This pilot study has some limitations: the presence of palpable lesions that were present may have had some impact in the surgeon’s decision to include additional margins that were found without clear correlation with the imaging or pathology outcomes. This type of bias may not be feasible to be removed in the clinical scenario that includes multiple surgeons and reflects the usual procedures. Another limitation of this study was not recording the time spent in performing the specimen sonogram in addition to the standard of care digital radiograph of the specimen. The mean time required for technique using immersion of the specimen on saline solution was reported as 5.2 min by a group from Korea (Lee et al. 2008). Therefore, we believe that the additional time for scanning the specimens is acceptable in a standard of care clinical practice.

In conclusion, specimen sonogram with high-resolution sonography associated to knowledge of imaging isocenter of targeted lesion demonstrates a promissory auxiliary tool to potentially select patients where re-excision is appropriately indicated and a valid component as one quality measure in breast conserving surgery.
